# Supplementary figures and images for: High-Throughput Tracking of Freely Moving Drosophila Reveals Variations in Aggression and Courtship Behaviors
Source: bioRxiv. 2025 Jul 13:2025.07.10.663947. Preprint. [Version 1] doi: 10.1101/2025.07.10.663947 (PMC12265614; doi:10.1101/2025.07.10.663947)

$\alpha$ - mCD8

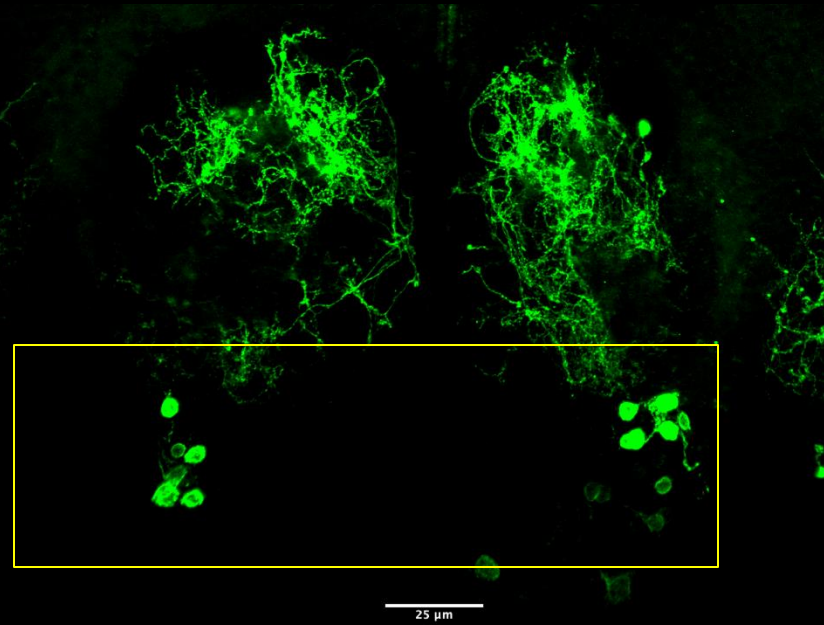

$\alpha$ - Tdc2

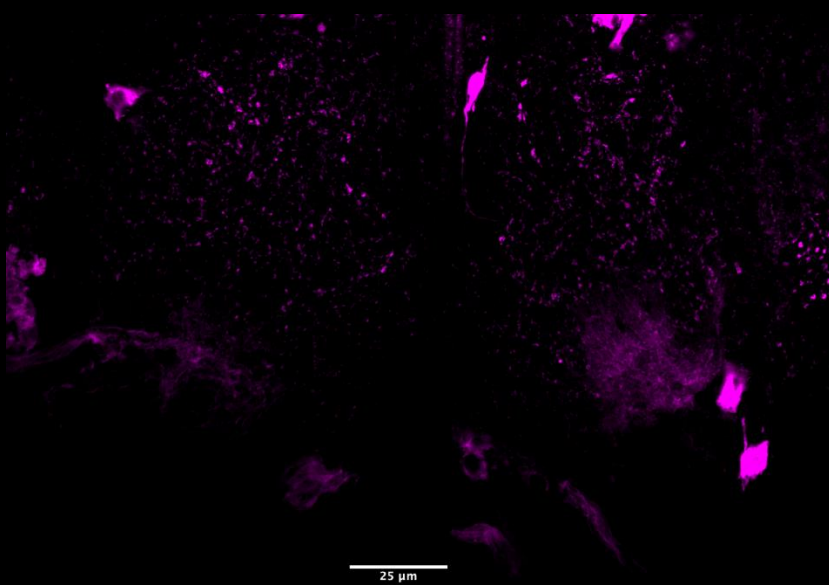

Merge

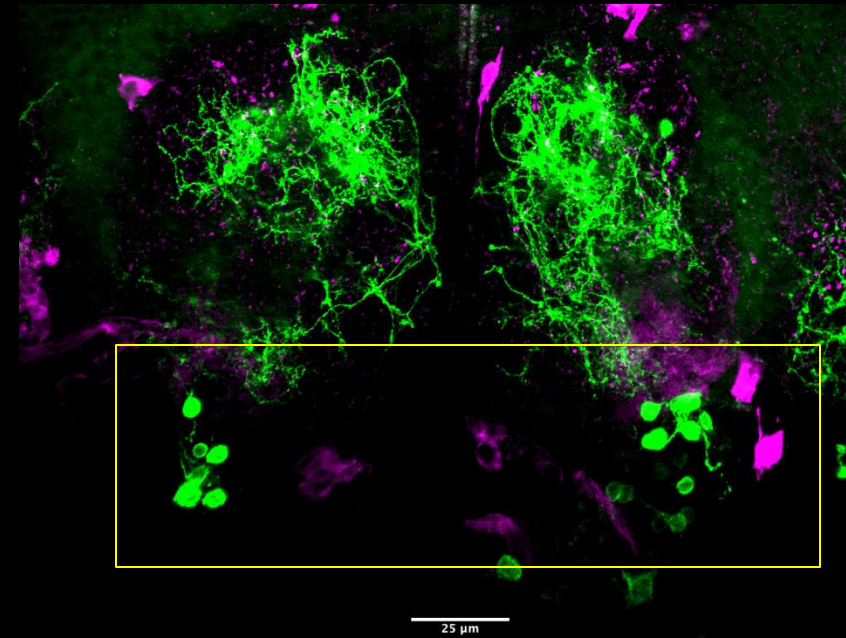

Supplement: Supplement 5 — Supplementary Figure 1 ∣ Confocal images showing higher magnification views of the R72A10-GAL4-labeled neuronal cluster at the AL–SOG region in a male adult Drosophila brain. The neuronal cluster (green, left) at the AL–SOG junction does not show co-localization with anti-Tdc2-positive neurons (magenta, middle) in the merged image (right), indicating that this cluster is not octopaminergic. Scale bars, 25 μm [file media-5.pdf]
